# Mitochondrial transfer in hematological malignancies

**DOI:** 10.1186/s40364-023-00529-x

**Published:** 2023-10-05

**Authors:** Xiaodong Guo, Can Can, Wancheng Liu, Yihong Wei, Xinyu Yang, Jinting Liu, Hexiao Jia, Wenbo Jia, Hanyang Wu, Daoxin Ma

**Affiliations:** Department of Hematology, Qilu Hospital of Shandong University, Jinan, 250012 Shandong P.R. China

**Keywords:** Mitochondrial transfer, Tunneling nanotubes, Extracellular vesicles, Extracellular mitochondria, Hematological malignancies

## Abstract

Mitochondria are energy-generated organelles and take an important part in biological metabolism. Mitochondria could be transferred between cells, which serves as a new intercellular communication. Mitochondrial transfer improves mitochondrial defects, restores the biological functions of recipient cells, and maintains the high metabolic requirements of tumor cells as well as drug resistance. In recent years, it has been reported mitochondrial transfer between cells of bone marrow microenvironment and hematological malignant cells play a critical role in the disease progression and resistance during chemotherapy. In this review, we discuss the patterns and mechanisms on mitochondrial transfer and their engagement in different pathophysiological contexts and outline the latest knowledge on intercellular transport of mitochondria in hematological malignancies. Besides, we briefly outline the drug resistance mechanisms caused by mitochondrial transfer in cells during chemotherapy. Our review demonstrates a theoretical basis for mitochondrial transfer as a prospective therapeutic target to increase the treatment efficiency in hematological malignancies and improve the prognosis of patients.

## Introduction

Mitochondria are highly dynamic double-membraned organelles found in the majority of eukaryotic cells [1]. They contain circular DNA and independently carry out cellular processes such as gene transcription and protein translation. Mitochondria play a crucial role in the cellular metabolic pathways, such as supporting cellular activities through generating ATP and controlling the production of nucleotides, cholesterol, and heme [2]. Aside from energy generation, they perform a variety of other functions such as controlling programmed cell death [3] and regulating cell proliferation. They enable interaction with other organelles, store calcium ions, and control the dynamic balance of cellular calcium ion concentration [4].

Mitochondrial malfunction has been involved in a broad spectrum of human illnesses. Since energy production is closely linked to mitochondria, mitochondrial dysfunction may be the initiating link in many diseases such as neuronal degeneration. For example, oxidative stress can lead to impairment of mitochondrial structure and function, and consequently synaptic damage and neuronal apoptosis, thus promoting the progression of Alzheimer’s disease [5]. Also, the imbalance in dynamic mitochondrial fission has been found in cardiovascular diseases such as atherosclerosis [6] and metabolic diseases [7].

Mitochondria were formerly assumed to be permanently housed in their somatic cell, however, recently they have been reported to be transported between cells [8–10]. This phenomenon is known as intercellular mitochondrial transfer and constitutes one of the eukaryotic cells’ innate survival systems. This is a novel mechanism for intercellular communication. More recently, increased evidence proved that mitochondrial transfer could occur between many cell lines. Mesenchymal stem cells (MSCs), for example, transport mitochondria to multiple cells, involving epithelial cells, macrophages, and tumor cells [11–13]. Another example is the discovery that macrophages could obtain mitochondria from nearby adipocytes, a process that identifies a transcriptionally different macrophage subpopulation [14]. Macrophages also act as donors to deliver mitochondria to cardiomyocytes by endocytosis, triggering ferroptosis and thus causing cardiomyocyte damage [15].

Mitochondrial intercellular transfer enhances mitochondrial integration to the endogenous web of recipient cells, leading to a significant change in the bio-energetic state and other functions in the receptor ones, and also generates changes associated with cell differentiation, cell survival, and even drug resistance [16]. Therefore, mitochondrial transfer has emerged as an excellent therapeutic strategy. Replacing nonfunctional mitochondria with healthy ones has the potential to revert mitochondrial malfunction in some mitochondrial diseases, thus restoring the bioenergy requirements of impaired cells [17]. Moreover, transport of healthy mitochondria rejuvenates the impaired cells like epithelial cells [18], neurons [19], and cardiomyocytes [20].

Recently, mitochondrial transfer has been observed in several kinds of hematological malignancies involving acute myeloid leukemia, acute lymphoblastic leukemia, and multiple myeloma. In these hematological diseases, mitochondrial transfer seems to promote tumor progression and result in chemotherapy resistance [20–22], implying that mitochondria make an appealing and biologically reasonable therapeutic target in hematological malignancies. Therefore, clarifying the functions and mechanisms of mitochondrial transfer would provide novel therapeutic approaches for leukemia and other diseases.

## Mechanisms of mitochondrial transfer

**Fig. 1:**
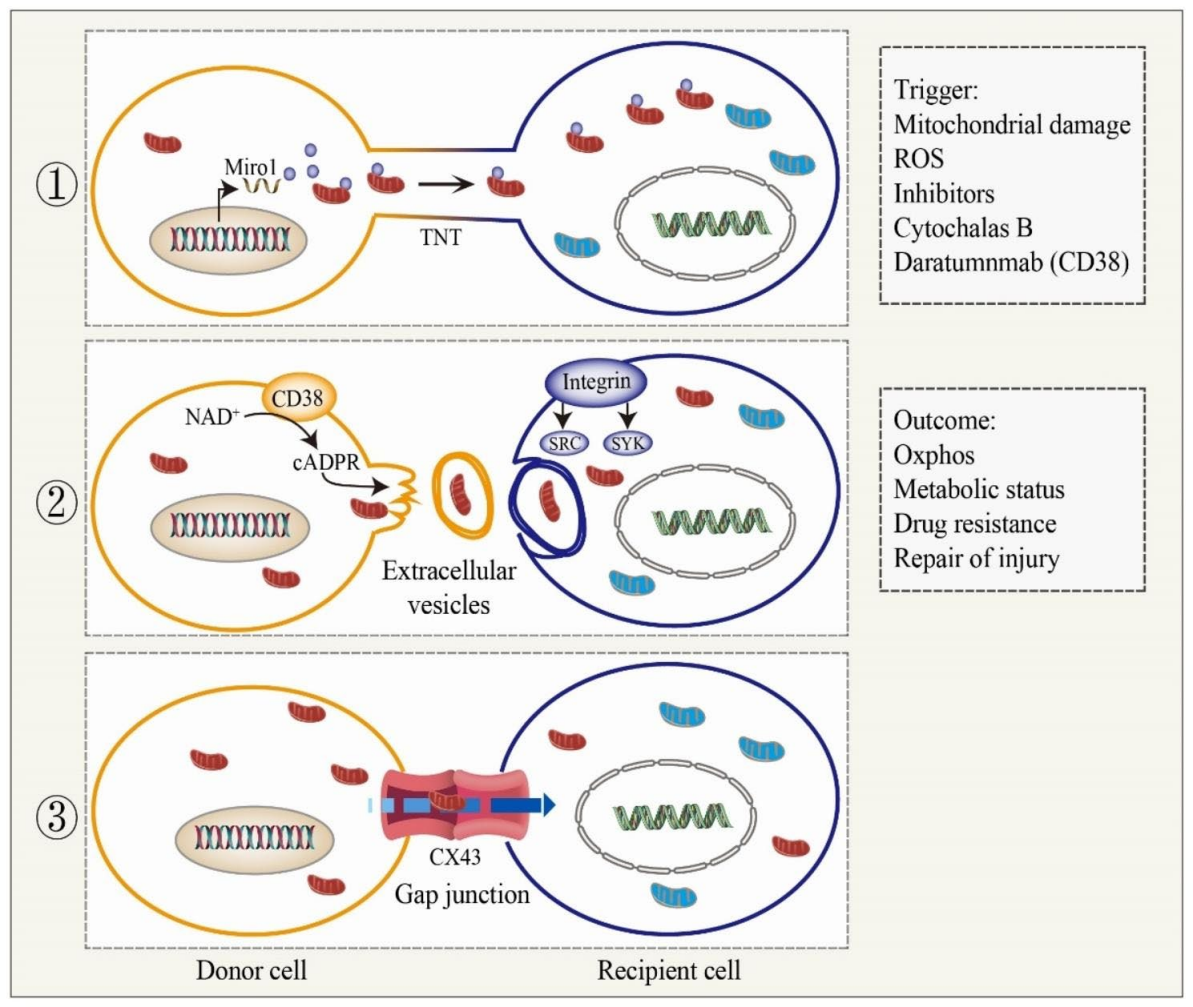
Three main mechanisms of mitochondrial transfer. The main influence factors and outcomes of TNTs, EVs and GJs are shown in the figure

There have been a few mechanisms described to mediate transcellular mitochondrial transfer. Among them, tunneling nanotubes (TNTs) are the primary means of mitochondrial transport, and multiple TNTs are able to be built between cells to interconnect, constituting a complex regulatory network for intercellular substance and signaling [23]. Other transfer mechanisms are already discovered, such as extracellular vesicles, gap junctions, and cell fusion (Fig. 1). Understanding the mechanisms which mediate mitochondrial transfer is of great importance to elucidate the regulatory processes and functional outcomes.

### TNTs

TNTs appear to represent a novel route of intercellular communication, indicating the ability to swap organelles for mammalian cells [8]. As is known, TNTs are open-ended tubes/channels that join the cytoplasm in two or as many interactive cells. TNTs transport lipid droplets, proteins, ions, RNAs, organelles, and pathogens bidirectionally over ‘long’ distances (150 mm) [8, 23, 24]. TNTs can be engaged in a variety of physiological and pathological events, like immune reactions, cellular proliferation and apoptosis, pathogen transport, and angiogenesis [25, 26]. To date, TNTs have been identified in different organisms and tissues, for example, MSCs [27], macrophages [28], neuronal cells [29], cardiomyocytes [30], T-cells [31], and renal proximal tubular epithelial cells [32].

#### Formation of TNTs

TNT formulation is mediated by two main pathways. The former mechanism relies extremely on cell mobility. When two cells approach each other spatially and then separate after close contact, the remaining membrane pores form TNTs [30, 32]. The process could be temporarily modulated since the continuous intercellular contact occurs within a few minutes. The second occurs when donor cells extend and integrate directly onto the cell membrane of target cells via membrane protrusions (MPs) that contain actin filaments, independent of cell fluidity or close contact. TNT generation, however, is relied on the actin protein in both methods.

TNTs have a cytoskeleton consisting mainly of cell membranes as well as fibrillar actin and microtubulin [33]. Besides fibrillar actin, there are also microtubules or cytokeratin filaments in TNTs of several cell lines [33–35]. These skeleton proteins aid in the active movement of cargoes and mitochondria between cells.

A number of molecular mediators of actin-driven TNT synthesis have been reported. [36–39]. Leukocyte-specific transcript 1(LST1, a small adaptor protein expressed in leukocytes [40]), for example, stimulates the development of TNTs via a RelA-reliant principle. When LST1 is recruited to the stromal membrane, it interacts with filamin by activated RelA (RelA-GTP) [39, 41]. In addition, a myeloid-specified protein, M-sec (a protein capable of modifying the cytoskeleton), is associated with the synthesis of thin TNTs [37]. It has been demonstrated that M-sec expression is required for TNT-mediated freight transport [36, 40, 42]. In reality, increased expression of M-sec stimulates the formulation of TNTs, while knock-out of M-sec reduces the production of TNTs by up to one-third [36, 43]. Nevertheless, the definite mechanism of its participation in TNT formation needs further investigation.

Several other pathways have been reported to affect the synthesis of TNTs. Under oxidative stress, the cellular expression of p53 is elevated and activation of AKT-PI3K-mTOR signal pathway is achieved, controlling the protrusion and growth of nanotubes [44]. Meanwhile, cytokinesis control protein 42 homolog (CDC 42) has also been proven to be essential in the elongation of TNTs after cell membrane bulging in donor cells [37].

#### Molecular actors of TNTs

Mitochondrial transfer is regulated by actin motors and Milton adaptor proteins [44, 45]. Miro1 protein (encoded by RHOT1) is an intracellular calcium-sensitive bridging protein concerning the modulation of mitochondria homeostasis and transmission. Miro1 binds mitochondria to KLF 5 kinesin under assistance of other auxiliary proteins such as TRAK 1, TRAK2 and Myo 10. They shape a motor adaptor complex when combined [20], thus facilitating mitochondrial transport in TNTs and regulating their motion. Meanwhile, these structures stabilize and preserve mitochondria against degradation [46, 47].

It is known that Miro1 functions in neurons as well as among other kinds of cells [48–51]. Miro1 plays a leading role in mitochondrial location and transfers in remote trafficking mediated by tubulin cytoskeleton [52]. When Miro1-overexpressied MSCs were co-cultured with injured epithelial cells, there was an enhanced development of TNTs between the two cells along with an increasing number of mitochondria transport [13]. The MSCs exhibited increased rescue potential, which contributed to the repair of epithelial damage. However, knockout of Miro1 with shRNA had the opposite effect, showing a significant decrease in mitochondrial donation [33]. All these data indicate that Miro1 expression regulates mitochondrial transfer [20].

Miro2 protein (encoded by RHOT2) is also the main connector for intracellular transport of mitochondria. In the presence of Miro1, it seems somewhat redundant for Miro2 in that it does not fully compensate for the loss of Miro1. Surprisingly, the two Miro proteins modulate kinesin-based motion, with Miro2 playing a dominating part in short-distance transmission [53] and more involved in modulating the connection of mitochondria and the actin cytoskeleton.

### Extracellular vesicles (EV)

In general, a diverse population of vesicles are discharged from the internal into extracellular environment, and the vesicles are named EVs. They are globular structures enclosed by a phospholipid bilayer membrane which encases many cellular proteins, nucleic acids, chemicals, and structural components [53–55]. Based on the source and molecular structure, EVs include exosomes (30–100 nm in diameter), microvesicles (MVs) (100 nm to 1 μm in diameter) or apoptotic bodies (50 nm-2 μm in diameter) [56]. Because of their quick removal by macrophages, apoptotic bodies are rarely examined [57].

EVs can carry multiple substances over long distance to alter the fate of host cells and organismal functions [56, 58]. Besides TNT-mediated transport, mitochondria could be packed in EVs [59, 60], and transferred between cells. The imported mitochondria can avoid lysosomal destruction after being endocytosed by the target cell and coexist with the new host cell. This is an effective method for moving functional loads from one cell to a different one [61, 62]. These findings add an innovative way of engagement to the multidimensional communication network, as well as a new signal transport mechanism [63].

Many types of cells, such as astrocytes and MSCs, can secrete mitochondria-carrying EVs [20, 63, 64] and transfer them to epithelial cells, immune cells and neurons [65]. It has shown that MSCs could modulate macrophages by EVs-containing mitochondria, enhancing their respiratory and phagocytic function in clinically related lung damage models [66]. Furthermore, another study reported that EVs from hormone therapy-resistant breast cancer patients contained high levels of mitochondrial DNA (mtDNA). MSCs, as the mesenchymal component of tumors, could transfer mitochondria via EVs towards neoplastic cells and promote the oxidative phosphorylation (OXPHOS), which facilitated cancer cell proliferation as well as metastasis; on another aspect, tumor-derived exosomes affected the differentiation of MSCs by regulating diverse signaling pathways [67]. It has been recently discovered that the release of microvesicles from astrocytes is controlled by a calcium-reliant mechanism concerning CD38 and circular ADP ribose signaling [64]. Via this progress, astrocytes transported their mitochondria to neurons affected by stroke as a “rescue” phenomenon. In the opposite case, EVs can be utilized to clear impaired-depolarized mitochondria from bone marrow mesenchymal stem cells (BMSCs) and be exported to nearby macrophages [68]. This transport recycled the mitochondria and secreted exosomes, and allowed macrophages to tolerate the transferred damaged mitochondria. Besides, another study showed that thermogenically stressed brown adipocytes prevent the defeat of the thermogenic procedures by releasing oxidatively damaged mitochondria via EVs. When reabsorbed into parental brown adipocytes, EVs containing mitochondria have been cleared by phagocytic activity of macrophages, which contributed to the protection of cellular physiology [69]. Mitochondrial uptake via EVs vectors has been described in various physiological states and diseases. Though CD38 is expressed on various types of leukemia including CLL [70] and MM [71], the specific role of CD38 in mitochondrial transport through EVs by leukemia cells in bone marrow microenvironment is unclear.

### Gap junctions (GJs)

GJs are another important ways for mitochondrial transfer, though there are fewer studies about GJs compared with TNTs due to that TNTs are easily observed in in vitro co-culture systems [20]. GJs consist of gap junction protein (connexin, Cx), connexon and gap junctional channels (GJCs), which join the cytoplasm of two separate cells and serve as an important channel for material exchange and signal communication between neighboring cells. The six identical Cxs on the cell membrane form a tubular structure around the linker. Two linkers on neighboring cell membrane connect end-to-end to form GJs. And the GJCs allow ions (Na^+^, K^+^, Ca^2+^, etc.), second messengers (e.g., IP3, cAMP, cGMP),and other small molecules to be exchanged across cells [72]. Therefore, GJs could regulate the intracellular mechanisms of signaling and autophagy. In vertebrate cells, Cx is a homologous transmembrane protein encoded by a multigene family. Over 20 Cx isoforms have already been defined, among which Cx43 is the most broadly expressed as well as thoroughly investigated. Cx43 is engaged in a range of physiological activities, for example, substance exchange, vesicle transport, and mitochondrial respiration [73]. Studies have shown that Cx43 is essential in mitochondrial uptake during lung and brain injury responses [73, 74] and also regulates CXCL12 excretion of MSCs [75].

In a study examining the protected effects of mitochondrial exchange of BMSCs in lung tissue, BMSCs release vesicles encapsulating mitochondria to reach alveolar epithelial cells via GJs, which are subsequently taken up by endocytosis [76]. This mitochondrial transfer mediated by Cx43 markedly reduced Cx43-mediated mitochondrial transfer markedly reduces LPS-induced acute lung damage, which includes enhanced leukocytosis, protein leakage, suppression of surfactant secretion, and high fatality rates [77]. Moreover, it has been demonstrated that GJs could mediate mitochondria transmission out of MSCs to the damaged motor neurons [78]. Also, Cx43 and Cx32 could create heterotypic GJs from MSCs to neurons, in which Cx43 was displayed on MSCs instead of motor neurons; whereas, Cx32 was expressed exactly the opposite way of Cx43^17^. GJs play important roles in mediating mitochondrial transfer via Cx43 and other proteins.

### Cell fusion

Except for TNTs, EVs and GJs, cell fusion is another proposed method of mitochondrial transport in cell-to-cell communication. As cell fusion is rarely found in higher eukaryotes under normal physiologic conditions, it is not the main mechanism for mitochondrial transfer.

Cell fusion is the process whereby the membranes of two or several cells unit together and partake organelles and cytoplasmic components. The process may be triggered when injury and inflammation occur [79]. Particularly, cell fusion modulates the stem cells potential and takes a crucial part in both regeneration and tumorigenesis [80]. Cell fusion is either transient or perpetual. After permanent cell fusion occurs, hybrid cells share cytoplasmic components and selectively lose donor cell nuclei, and recipient cell nuclei are reprogrammed to exhibit tissue-specific stem/progenitor cell properties [81]. As for transient cell fusion, it permits a temporary intercellular exchange of substances and signals, including transfer of donor cell mitochondria [82]. Fusion of MSCs and terminally differentiated somatic cells mediates reprogramming of the latter and contributes to tissue regeneration [83].

As for the form of cell fusion, it occurs both by partial cell fusion formed by TNTs and by full cell fusion. When co-cultured human MSC with transgenic mouse cardiomyocytes, the transfer of mitochondria can be identified at full fusion of MSCs with cardiomyoblasts with mitochondrial damage [84]. Yet, mitochondria have as well been shown to be transported through TNTs between the two cells, enabling cells in a state of oxidative stress to exchange damaged mtDNA during fusion and maintain the mitochondrial biogenesis of aerobic respiration. Likewise, primary glioblastoma cells acquired mitochondria through phagocytosis of tumor-activated stromal cells (TASC), extracting TASC cytosolic components for themselves [85]. Therefore, mitochondrial transfer could be mediated by cell fusion in physiological and pathological states.

## Trigger signals

### Early signals triggering mitochondrial transfer

Stress conditions, like infections or inflammations, hypoxia, x-rays, and ultraviolet, can trigger early signals for mitochondrial transfer [27, 33, 85–91]. Stressed cells emit early stimulation signals, and donor cells receive them from the local microenvironment and then undergo massive synthesis of mitochondria, which select different pathways for transfer in a specific environment. When severe tissue injury occurs, many mitochondria-related components including mtDNA and extracellular ATP are liberated outside the injured cells in the form of damage associated molecular patterns(DAMPs), which accumulate around the injured tissue or cross through capillaries and merge into the bloodstream [92, 93]. Besides DAMPs, reactive oxygen species(ROS) from cells in stress or inflammatory states also stimulates mitochondrial trafficking from donor to recipient cells [13]. By releasing high levels of ROS as a distress signal, stressed cells acquire mitochondria from other cells so as to down-regulate intracellular oxidative stress [94]. Moreover, factors that govern mitochondrial transfer include mitochondrial autophagy [94, 95], KIF5B, glucose [96], ATP [65], TNF-α [12], and the microenvironment of BMSCs.

Functionally normal mitochondria are able to be transmitted from MSCs into receptor cells, while injured mitochondria in recipient cells under stress can also be transferred to MSCs via TNTs and be cleared by mitophagy [95]. In this case, mitochondria of damaged cells work as danger-signaling organelles [97]. Through the bidirectional transfer of TNTs, stressed cells can also transfer substances such as ROS to MSCs, as well as retrograde signaling like AMP/ATP and NAD+/NADH in stressed cells [20]. On the one hand, the retrograde signaling in TNTs acts as an early stimulatory signal to stimulate mitochondrial biosynthesis and transfer within MSCs via upregulating the expression level of the protein PGC-1α related to mitochondrial biosynthesis [98]. On the other hand, it can stimulate the function of MSCs against apoptosis and cell damage repair [99]. Therefore, it is possible to avoid apoptosis and promote cell survival after receiving stress signals and transferring components such as mitochondria. In this light, TNTs might be viewed as a facility for increasing cell survival under stress [100].

### Nuclear factor kappa B (NF-κB) signaling

NF-κB signaling presents in most cells, and is involved in inflammatory and other diseases [101]. Dysregulated NF-κB signaling causes chronic inflammation and autoimmune diseases. TNF-α-NF-κB-TNFaIP2 signaling pathway has been found to be engaged in TNT synthesis among MSCs and cardiomyocytes, indicating inflammation is essential to influence the efficiencies of mitochondrial transfer in MSCs [12]. TNF-α could activate NF-κB pathway and undergo phosphorylation, thereby stimulating the expression of TNFaIP2 protein. The upregulated protein provokes the accumulation of F-actin, resulting in improved mitochondrial transport between MSCs and impaired cells. Sc-514, an NF-κB inhibitor, greatly lowers TNT generation, indicating that NF-κB signaling regulates TNT synthesis [12]. In addition, another study also discovered that NF-κB activation participated in the modulation and synthesis of TNTs, while cytarabine alone or in combination with daunorubicin inhibited NF-κB and downregulated TNTs [102]. All these data offer further support to the NF-κB pathway to participate in TNT formulation.

## Extracellular mitochondria

Although most studies demonstrated that mitochondrial transfer occurs via intercellular mechanisms, more and more evidence indicate that mitochondria could be freed to the extracellular environment and then transmitted to donor cells [103]. As previously noted, mitochondria could be exited in extracellular environment in integral and free form (freeMitos), or be surrounded with membranes, like within vesicles, or as cell-free circulating mtDNA (ccf-mtDNA) [104]. These various kinds of mitochondria perform diverse roles varying from promoting restoration effects to serving as signals on interaction with other cells.

It has been proven that cells could leave some elastic fibers behind during migration, and little vesicles grow at the top or intersection of the elastic fiber, which are named migrasomes [105]. During cell migration, some intracellular material is continuously transported to the migratory body by constricting the duct of these fibers. And then the contracted fibers are broken, leading to the release of migrators and engulfment by cells around the extracellular space. Furthermore, mitochondria were unexpectedly found present in extracellular migrators, and these mitochondria showed an unhealthy and damaged state. Overall, the study discovered a previously unknown phenomenon in cells, mitocytosis (mitochondrial exocytosis), which could clean out damaged mitochondria and maintain mitochondrial homeostasis in cells.

These external mitochondrial interactions with other cells open up a novel field of research where mitochondria transcend the roles as cellular powerhouses and act as signal organelles [105–107]. Elucidating the effects of extracellular mitochondria and their various formats helps develop new therapies for better health and define innovative disease-related biomarkers.

## Biological functions

Mitochondrial transfer may preserve metabolic homeostasis and cell-to-cell interaction in the microenvironment in response to metabolic and oxidative stressors. It alters the functional status of recipient cells, and takes a critical part in the progression and viability of neoplastic cells as well as in the repair of damaged cells.

### Metabolic communication

Through TNT-mediated mitochondrial transport, the energy metabolism of recipient cells has been changed, with the increased production of OXPHOS and ATP and thereby maintaining metabolic homeostasis in a variety of cells [108].

As is reported, mitochondrial transmission from MSCs to adenocarcinoma cells has caused a series of changes of the latter in energy metabolism, including decreased extracellular lactate and ROS, and increase in extracellular ATP, membrane potential and oxygen consumption, indicating a complete recovery of mitochondrial activity after being co-cultured. Similarly, osteosarcoma cells, after co-culture with MSCs, displayed the higher mitochondrial activity by boosting intracellular ATP and oxygen consumption rates [109]. Also, damaged astrocytes can receive mitochondria of MSCs, thus restoring their energy productivity and cell proliferation [110]. The heat-stress-producing brown adipocytes are demonstrated to release EVs containing oxidatively damaged mitochondrial fractions and are cleared by macrophages, thereby maintaining thermogenesis as well as cellular metabolic homeostasis [69]. It is also shown that adipocytes transport intracellular mitochondria to macrophages to regulate the homeostasis of white adipose tissue and could be inhibited in obese conditions [14]. In a preclinical acute respiratory distress syndrome model, evidence suggests that mitochondrial transport from MSCs towards macrophages takes a critical part in immune response [111]. And, direct co-culture of MSCs with phagocytic cells enhances the OXPHOS and intracellular ATP activity of recipient cells, and then enhances their phagocytic activity and immune response, thus promoting the improvement of the repair process.

### Regulation of tumor microenvironment and chemo-resistance

The progression of tumors requires interaction between tumor cells and neoplastic microenvironments. In normal conditions, mitochondrial transfer can induce cellular reprogramming. It has been demonstrated that co-culture of fully-differentiated cardiomyocytes and MSCs caused the mitochondrial transfer from MSCs into cardiomyocytes, promoting a progenitor-like state of the latter [82]. As for cancers, tumor cells can motivate stromal cells to increase the range of pathways that support cancer cell proliferation, hence promoting tumor growth. It has been found that a vital molecular signal could be transmitted from tumor cells to stromal cells by TNTs, causing consequent generation of survival-promoting molecules including cytokines [112]. Moreover, mitochondrial transfer may facilitate metabolic reprogramming of tumor [113]. Tumor cells utilize mitochondrial transport to reconstitute the tumor microenvironment, resulting in enhanced intracellular mitochondrial function, thereby causing an elevated proliferation and aggressive phenotype of these cells. For example, in the bladder cancer model, the invasion of tumor cells has been enhanced in vitro and in vivo by mitochondria transport [114].

Meanwhile, mitochondrial transmission has been shown to mediate chemotherapy resistance [115] in many tumors including breast, ovarian, and bladder cancer. Under co-cultured conditions, the breast or ovarian cancer model indicated the presence of TNTs between tumor cells and BMSCs. Mostly, mitochondria migrated from BMSCs to cancer cells, enhancing the chemo-resistance to the DNA-damaging drug doxorubicin [10]. Besides, after transferring healthy mitochondria into breast cancer cells with dysfunctional mitochondria, normal aerobic respiration of cancer cells was restored and cell invasiveness was enhanced [116]. However, the opposite result is observed in artificial-isolated mitochondria transferred from normal breast epithelial to breast cancer cells [117]. The viability and tumorigenicity of the tumor cells have been impaired as a result of mitochondrial transplantation related apoptosis mediated by AIF, the increased parkin protein as well as reduced fragmented mitochondria [118].

### Tissue repair

Mitochondrial transfer could promote wound healing and might be implemented in the repair of impaired tissue. Mitochondrial transfer has been proven to take a protective part in the vascular system. Mitochondria transferred between endothelial progenitor cells via a TNT pattern could promote regeneration and repair of damaged myocardium [119]. In addition, when co-culture human adipose stem cells and murine cardiomyocytes, it can be found that the intercellular F-actin junctions were formed which was associated with mitochondrial transfer [82]. The transferred mitochondria are helpful in the recovery of cellular status through partial cell fusion. Another study transplanted BMSCs to rats via the carotid artery, and the results suggested the intercellular mitochondrial transmission between MSCs and injured endothelial cells prevented oxidative damage and apoptosis under cerebral ischemia-reperfusion conditions [120], further confirming its protective role in the damaged cerebral microvascular system.

Intercellular mitochondrial transfer is also reported to be involved in the maintenance of homeostatic of lung tissue. Mitochondrial transfer was observed from BMSCs to alveolar epithelial cells in a murine model of acute lung damage treated with LPS, and resulted in increased alveolar ATP levels [76]. This mitochondrial transfer exerted a protective effect on alveolar epithelial cells, thereby repairing airway damage and improving lung inflammation [33]. In astrocyte/neuronal co-culture systems, neurons damaged by rotenone were related to reversal of neurodegeneration and axonal pruning after internalization of mitochondria [121]. In the murine model of transitory focal cerebral ischemia, healthy mitochondria were released from astrocytes and absorbed by neurons, and restored their aerobic cellular respiration and proliferation [110]. These discoveries indicate an effect for mitochondrial transfer in neurological recovery after stroke, involved in the maintenance of neural integrity, with potential in the treatment of neurodegenerative diseases.

In serious corneal injury situations, corneal stem cell transplantation has become an emerging tactic for corneal regeneration and scar formation prevention. The rate of bioenergetic parameters such as ATP production and maximal respiration rate were inferior in rotenone-treated corneal epithelial cells in comparison to healthy corneal epithelial cells. All these indices showed significant improvement in corneal epithelial cells co-cultured with MSCs, suggesting that mitochondrial transmission in MSCs is a crucial mechanism of MSC treatment for corneal impairment [13]. In addition, when co-cultured BMSCs with renal tubular epithelial cells under high-glucose exposure in a diabetic nephropathy model, the results showed that BMSCs imported their mitochondria into injured renal proximal tubular epithelial cells by TNTs, which significantly inhibited the apoptosis of the latter [18]. All these data show that mitochondrial transfer is an important and novel strategy for tissue repair.

## Mitochondrial transfer in hematopoiesis and hematological malignancies

### Hematopoiesis

Mitochondrial transfer has been observed in normal hematopoiesis. Upon exposure to bacterial infection, hematopoietic stem cells (HSCs) expanded quickly in response to stress stimulation and the immune system underwent a rapid granulocytic response associated with acute bacterial infection. At this time, mitochondria were transferred from BMSCs to HSCs via GJs under the regulation of ROS. As a result, the mitochondrial mass of HSCs increased, leading to a metabolic shift from glycolysis to OXPHOS. Mechanistically, ROS-induced oxidative stress regulated phosphatidylinositol 3-kinase (PI3K) activation to mediate the opening of connexin channels, thereby permitting transport to occur. Here, HSCs act as recipients of transfer to acquire mitochondria in response to acute bacterial infection [21].

In another discovery, mitochondrial transfer was found to occur in the opposite direction, i.e., from hematopoietic stem and progenitor cells (HSPCs) to MSCs. After radiation prior to bone marrow transplantation (BMT), the mitochondria of MSCs were dramatically impaired. HSPCs transferred healthy mitochondria to the stromal microenvironment (ME) via HSPC Cx43-mediated cell contact, thereby improving mitochondrial activity in recipient MSCs. As a result, supportive stromal ME was restored and hematopoietic compartment reconstruction was improved. Understanding the mechanisms that regulate stromal recovery after myeloablative stress is highly clinically relevant for optimizing BMT procedures and emphasizing the importance of adjuvant non-HSC accelerated hematopoietic transplantation [122].

Moreover, after injecting erythroblast island (EBI) macrophages into mice that suffered from multiple modes of anemia stress, it could be found that mitochondrial transfer occurred from EBI macrophages to early erythroblasts via direct uptake. At the early erythropoietic stage, mitochondrial transfer alters the bioenergetic profile of recipient cells through CD47-Sirpα interactions, promoting their proliferation, protein synthesis, and energy generation, thereby driving the recovery of erythroid lineage cells in response to stress. This finding provides evidence for a supportive role of splenic and bone marrow EBI macrophages in erythropoiesis during erythrocyte stress and confirms the essential mediating role of mitochondrial transfer [123].

These above findings suggest that mitochondrial transfer occurs in normal hematopoiesis under infection, radiation and stress, and involves various cell types and different directions of transport. These transfers alter the metabolic state and physiological functions of the recipient cells, and facilitate the body’s adaptation to and recovery from stress.

### Hematological malignancies

**Fig. 2:**
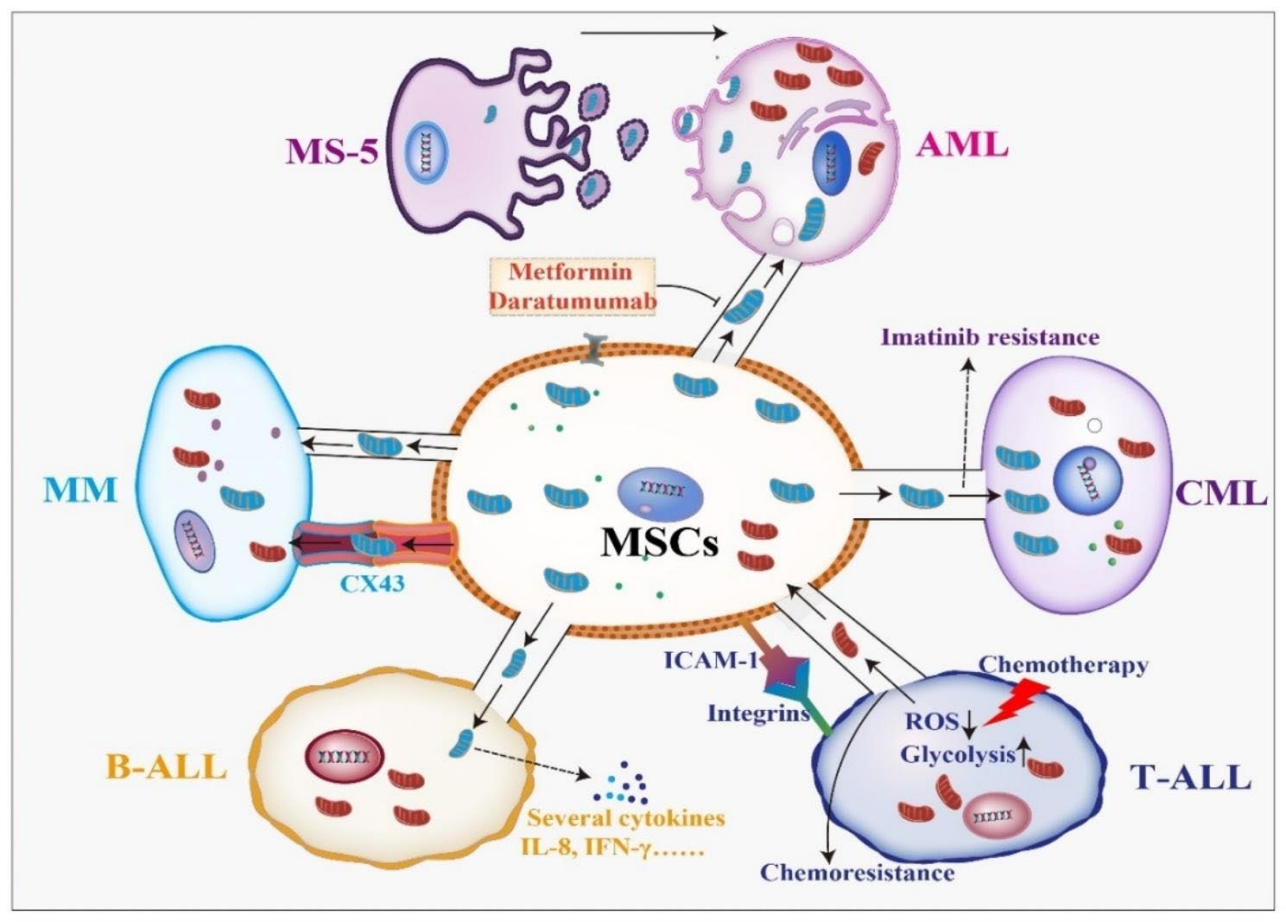
Mitochondrial transfer in hematological malignancies. Mitochondria are transmitted mainly by TNTs and other forms, leading an increase in survival of hematopoietic malignant cells and chemoresistance to drugs. More details can be seen in the text

Hematological malignancies define a highly heterogeneous disorder with strongly variant prognoses that continuously relapse after therapies. Hematological malignancies have the leading morbidity and mortality and seriously affect human health [124]. Because of the crucial role of TNTs in communication between cancer cells and BMSCs, mitochondrial transfer is also essential in hematological tumors [125]. The majority of researches on the contribution of mitochondrial transfer in hematological malignancies focus on leukemia and myeloma (Fig. 2).

#### Acute myeloid leukemia (AML)

AML is featured by the invasion of clonal and inadequately differentiated hematopoietic cells in bone marrow [126]. Via Warburg effect, a majority of tumor cells convert their ATP production from OXPHOS to glycolysis [127]. In contrast with the Warburg hypothesis, the survival of AML cells is strongly reliant on oxidative phosphorylation for ATP production, and they are constantly adjusting to variations of nutrition and oxygen supply in BM microenvironment [127, 128]. It has been shown that mitochondria and OXPHOS could affect chemotherapeutic drug sensitivity and efficacy in vivo. High OXPHOS signaling and metabolism were identified as key marker of chemo-resistance, and AML cells with this profile were more resistant to Ara-C chemotherapy. Similarly, essential mitochondrial function also contributes to increased AML resistance to Ara-C. Chemo-resistant leukemia cells could initiate tumor regeneration in vivo, exhibiting high OXPHOS genetic characteristics, high ROS levels with an altered intracellular redox state, and maintenance of polarized mitochondria [129].

It has been found that the proteins associated with cell energy metabolism are up-regulated in AML cells after cocultured with BMSCs [130]. Despite the improved metabolism in recipient cells, there is no definitive evidence that mitochondria are indeed transmitted. Moschoi et al. recently demonstrated that MS-5 unidirectionally transferred intact functional mitochondria into AML cells via the endocytic pathway and that mitochondrial transfer increased in a time-dependent manner, increasing ATP production by OXPHOS in AML cells up to 1.5-fold. The endocytosis inhibitors blocked mitochondrial transfer between murine MS-5 and human AML cells. During this process, AML cells became less sensitive to the chemotherapeutic drug Ara-C and the uptake of mitochondria was enhanced under chemotherapeutic conditions [131].

Mitochondrial exchange between BMSCs and AML cells has already been observed in co-culture conditions both by lentiviral transfection and fluorescent staining of BMSCs mitochondria, which was also shown in vivo [22]. A high level of oxidative stress has been found to exist in AML [132, which promotes ROS-driving mitochondrial trafficking from BMSCs to AML cells through TNTs. Specifically, NOX-2-derived superoxide produced from AML cells induces mitochondrial transfer. The antioxidant N-acetylcysteine (NAC) inhibited mitochondrial transfer, while the oxidant hydrogen peroxide (H2O2) further promoted mitochondrial trafficking between BMSCs and AML cells [22]. The elevated intracellular ROS levels in AML in response to chemotherapeutic agents further increased the oxidative stress environment, thereby increasing mitochondrial transfer. Suppression of NOX2 inhibited mitochondrial transport, enhanced AML apoptosis and increased the viability in an in vivo xenograft model. It suggests a unique treatment method for AML that involves tumor-specific reliance on NOX2-drived mitochondrial transfer. However, NOX2 inhibitors are not yet available in clinical trials. In addition, toxicity may be an issue with this strategy, as NOX2 deficiency in humans often ends in death within the first decade of life [133].

As mentioned before, AML cells rely on OXPHOS for ATP production. Another study explored the mechanisms whereby the bone marrow microenvironment promotes secondary resistance of AML cells to OXPHOS repression. They utilized IACS-010759 to treat AML cells, a newly complex I OXPHOS inhibitor, and discovered that direct contact with BMSCs induced the compensation of AML cells by activating mitochondrial respiration and developing tolerance to OXPHOS suppression. In terms of mechanism, suppression of OXPHOS inspired mitochondrial transport from MSCs towards AML cells through TNTs under direct-contact co-culture circumstances. Additionally, in AML cells, this inhibition induced mitochondrial division, an increase in functional mitochondria, and mitochondrial phagocytosis. Moreover, it was demonstrated that mitochondrial transmission of MSCs to AML cells induced by OXPHOS suppression was further strengthened by Ara-C [134]. These findings suggest that AML cells managed to replete functional mitochondria via exogenous transport of MSCs-provided mitochondria and internal fission of healthy mitochondria under OXPHOS restriction. Meanwhile, mitochondrial phagocytosis eliminates damaged mitochondria. These alterations have a major part within the compensatory adjustment of leukemic cells towards the energy stress of bone marrow microenvironment.

Clinical observational trials using Venetoclax combined with hypomethylating agents targeting BCL2, an important modulator in mitochondrial apoptosis pathway, have shown a tolerable safety profile and good overall remission rates in elderly AML patients [135]. Therefore, mitochondria are an appealing and biologically reasonable drug target for AML therapy. Through the utilization of an in vitro co-culture system and an in vivo mouse model, daratumumab, a monoclonal anti-CD38 antibody, has been found to impair the metabolic capacity of AML by blocking mitochondrial transport between BMSCs and the parent cells [136]. This leads to a decrease in AML-derived OXPHOS, thereby inhibiting tumor proliferation and reducing tumor load. Daratumumab should be investigated more as a treatment for mitochondria-dependent tumor development, according to these findings. Another study demonstrated that metformin, the most common medicine for curing type II diabetes, dramatically increased the chemo-sensitivity of AML cells under co-culture with BMSCs by inhibiting mitochondrial transport. Metformin’s chemo-sensitizing impact is mechanistically mediated by inhibiting intercellular contact-dependent mitochondrial transport and mitochondrial OXPHOS in receiver AML cells, with effects comparable to those of the control medicine cytochalasin D, a TNT formation inhibitor. Besides, metformin enhanced the anti-tumor effects of Ara-C in AML cells co-cultured with BMSCs in an immune-deficient mouse xenograft model [137]. This research determines the possible applicability of metformin in the sensitization of AML cells to chemotherapy. Metformin has been used in combination with a few anti-leukemia agents and shown positive therapeutic results. For example, through metformin-triggered mitochondrial membrane depolarization, the Bcl-2 inhibitor ABT-737 enhanced mitochondrial death of leukemic cells [138].

Current studies demonstrate that mitochondria transfer between AML cells and BMSCs via TNTs or endocytic pathways. AML receptor cells have enhanced metabolic capacity and resistance to chemotherapeutic agents. Mitochondrial transfer become a promising target in AML therapy. In combination with existing chemotherapeutic regimens, it can increase the killing power of chemotherapeutic agents on AML cells by inhibiting mitochondrial transport, which is expected to be widely applied in the clinic.

#### Chronic myeloid leukemia (CML)

CML is a myeloproliferative neoplasm and manifests increased peripheral blood leukocytes. CML is triggered by reciprocal chromosome translocations, which results in a BCR-ABL fusion gene [139]. The annual mortality rate has fallen dramatically [140] since the availability of imatinib [141]. However, some patients fail to obtain an entire cytogenetic response or acquire resistance and progression to a primitive stage. Stromal cells and leukemic cells are found to interact bidirectionally to support leukemogenesis [142]. Leukemia cells cultivated in vitro generally cease to exhibit drug-resistant characteristics, implying a cytoprotective role for the bone marrow microenvironment. In general, intercellular interaction is critical in stroma-mediated drug resistance in CML. The TNT formulation between CML cells and BMSCs facilitates the transmission of mitochondria, cellular vesicles and proteins [143]. It has been found that TNT bulges areas contained many vesicles, which were transmitted between stromal cells and CML cells via TNTs. TNT-mediated vesicles transport and functional proteome were involved in stromal protection of leukemic cells and contributed to survival of leukemic cells. As mentioned above, mitochondrial transfer can increase the resistance of CML cells to certain chemotherapeutic agents, thus protecting the leukemic primitive cells. Similarly, the transport of cell vesicles between stromal cells and leukemic cells was found to protect CML cells against imatinib-induced apoptosis, which increased the chemotherapy resistance of CML cells during imatinib treatment.

In conclusion, mitochondrial trafficking between CML cells and BMSCs by TNTs increases resistance towards chemotherapeutic drugs and decreases the apoptosis rate of CML cells. This could be considered a therapeutic tool when leukemic cells develop drug resistance or when individuals fail to obtain a complete cytogenetic response.

#### Acute lymphoblastic leukemia (ALL)

##### T-cell acute lymphoblastic leukemia (T-ALL)

T-ALL is one of the most invasive hematologic malignancies. It originates from the malignant conversion of T-cell progenitors. The cure rate of T-ALL has improved to about 50% in adults with the treatment of high-dose multi-agent chemotherapy [144]. However, lots of T-ALL patients develop primary chemo-resistance and leukemia recurrence. These difficulties continue to be the main obstacles to clinical attempts at curing T-ALL [145].

BMSCs have been shown to have an impact on cellular mitochondrial dynamics of T-ALL and affect the chemo-resistance of leukemia cells. In a study, MSCs were shown to provide protection against chemotherapeutic cell mortality and cytotoxicity in T-ALL under both indirect and direct co-culture conditions [146]. The cells in the direct contact system were more viable and had better pro-survival effects. Considering that mitochondria are a vital resource of ROS, upregulation of mitochondrial ROS level is a viable method to kill cancer cells [147]. And exposing of T-ALL cells to MSCs reduced the mitochondrial ROS level and facilitated pre-glycolytic transfer, which resulted in higher glucose absorption and lactate generation [146]. These findings suggest MSCs could preserve T-ALL cells from chemotherapeutic cytotoxicity and promote ALL cell proliferation and survival, which partly depends on reduced ROS contents in mitochondria as well as a pre-glycolytic metabolic transition. Furthermore, the protective actions couple with a mitochondrial breakage process that is controlled by ERK-mediated activation of DRP1 phosphorylation. Therefore, disrupting leukemic cell/matrix connections and focusing on mitochondrial dynamics could offer an innovative approach for T-ALL treatment that can be employed in conjunction with standard chemotherapeutic drugs. Moreover, it was proposed that T-ALL cells are capable to deliver more mitochondria to MSCs after chemotherapeutic drug-induced oxidative stress, but gain fewer mitochondria from MSCs. The process is medicated by TNTs and ICAM-1, which is helpful for chemo-resistance mediated by cell adhesion [148]. There are many examples of mitochondrial transport from MSCs to others, such as AML cells, breast cancer cells and other malignant cells. However, few have demonstrated mitochondrial trafficking from other cells to MSCs. The divergence in the direction of transfer might be due to their distinct states of metabolism. AML cells have more OXPHOS, while T-ALL cells are more glycolysis upon co-culture [149]. AML cells input mitochondria to meet the need for high-energy metabolism, whereas ALL cells expel mitochondria to decrease cellular ROS. These findings facilitate revealing the signals and mechanisms driving the directionality of transport. However, whether T-ALL cells might also provide mitochondria towards other cells, and what effect this has on tumor progression deserve further exploration.

The important role of mitochondrial metabolism in the maintenance of bioenergetic and metabolic homeostasis makes it an interesting therapeutic target. T-ALL cells have oxidative stress and metabolic disruption, which is a frequent hallmark of cancer cells. The level of ROS in T-ALL cells is much higher than that in normal cells. As excessive ROS causes leukemic cell death, it has been shown that inducing intracellular oxidative stress is an essential anticancer machinery of leukemia chemotherapy [150]. ALL cells display a hyperenergetic profile with high glycolysis and high OXPHOS [151]. Calcium migration to the mitochondria via the inositol 1,4,5-triphosphate receptor (InsP3R) maintains mitochondrial activity, which makes T-ALL cells susceptible to the suppression of InsP3R. Recently, after treatment by Xestospongin B (XeB), the specific inhibitor of InsP3R, higher mitochondrial respiration exhibited by T-ALL cells was attenuated [152]. Long-term therapy with XeB resulted in T-ALL cell death without influencing the normal counterparts. Therefore, inhibition of InsP3R by XeB has been considered as a potential therapy for T-ALL.

In summary, mitochondrial transport from T-ALL cells to MSCs is mainly regulated by TNTs, which reduces the level of mitochondrial ROS in the cells and induces drug resistance. Clinical disruption of the communication between leukemia and stromal cells could provide a novel therapeutic method in combination with conventional chemotherapeutic agents.

##### **B-cell acute lymphoblastic leukemia (B-ALL)**

Mitochondrial transfer is also present in B-ALL. It has been shown that primary B-cell precursor ALL (BCP-ALL) cells utilize TNTs to communicate with MSCs. The signaling led to the secreting of pro-survival cytokines, induced stroma-mediated prednisolone resistance, and increased the viability of B-ALL cells. Suppression of TNTs significantly inhibited this process and made BCP-ALL cells re-sensitized to a vital anti-leukemic medicine prednisolone. The discovery of TNT signals in ALL-MSC interaction sheds light on the pathobiology of ALL and brings novel opportunities for developing more efficient treatments [112]. Another study found that isolated MSCs from ALL patients undergoing chemotherapy are typically activated, and carry cancer-associated fibroblast (CAF) phenotype. The phenotype has an altered cytoskeleton and gene expression with high levels of pro-inflammatory cytokine secretion. A subsequent series of experiments demonstrated that primary MSCs and MSC cell line HS27a could be triggered into CAF by clinically relevant concentrations of Ara-C and erythromycin. CAF/activated MSCs transferred mitochondria into B-ALL cells via TNTs, which prevents exogenous ROS-inducing agent-induced B-ALL cells from apoptosis and death [153]. Overall, mitochondrial trafficking decreases the sensibility of B-ALL cells to chemotherapy treatment and protects B-ALL cells from ROS-induced cell death. Moreover, mitochondrial transfer contributes to ALL cells survival and promotes ALL development, and the specific mechanism remains unclear.

#### Multiple myeloma (MM)

MM is a hematological malignant tumor that features abnormal clonal plasma cells in bone marrow [154]. The growth of abnormal clonal plasma cells causes damaging bone lesions, acute kidney impairment, anemia and hypercalcemia [155]. The disease generally progresses slowly and remains a malignancy that cannot be completely cured.

It is recognized that MM cells typically utilize a non-mitochondria-based glycolytic process to produce their ATP and be sensitive to glycolytic inhibitors [156]. The dependence of MM cells upon oxidative phosphorylation is triggered by an intercellular transport of mitochondria from adjacent nonmalignant BMSCs to increase cellular respiration, leading to increased proliferation of MM cells [157]. CD38, a typical marker of MM cells, has been linked to mitochondrial transfer medicated by TNTs. For example, the non-malignant transmission of mitochondria from astrocytes to neurons after stroke is governed by CD38^64^. In MM cells, CD38 was shown to be highly expressed [71], while shRNA-mediated knock-down of CD38 inhibited mitochondrial transport to MM cells and improved survival in animals in vivo. Inhibition of CD38 is a viable therapeutic strategy in MM in the current clinical setting, where treatment combination using drugs targeting CD38 such as daratumumab has shown considerable clinical effect in previously untreated and relapsed MM patients [158]. Thus, these studies provide a scientific basis for the inhibition of mitochondrial transport in MM, and a physiological basis for the choice of proper drugs to be applied in conjunction with mitochondrial transport blockers.

It was reported that myeloma MSCs are less dependent upon mitochondrial metabolism than healthy ones, exhibiting significantly higher glycolytic rates such as decreased NAD+/NADH ratios and increased intracellular lactate [159]. The metabolic reorganization of MM-MSCs may rely upon the hypoxic BM milieu, while MSCs also showed an enhanced proclivity to transport mitochondria to MM cells. This suggests that mitochondrial transport between plasma cells and stromal cells could contribute to the pro-tumor phenotype of MSCs. CXCL12/CXCR4 axis, which is important for normal and MM cells homing in the bone marrow, has been reported to be related to mitochondrial transmission [160]. CXCL12 is a critical modulator in the tumor microenvironment, influencing various oncogenic processes including angiogenesis, osteoclast-genesis, tumor cell immigration and adherence to stromal cells [161]. Furthermore, it was also found that plasma cells promoted CX43 expression of MSCs, leading to the activation of CXCL12 and its receptor CXCR4 on MM cells, which facilitates mitochondrial trafficking between MSCs and plasma cells. The selective suppression of CXCR4 by Plerixafor contributed to a considerable reduction in mitochondrial transport. Furthermore, CXCR4 intracellular expression in myeloma cells from BM specimens showed more co-localization with CD138 + cells in bortezomib-naive patients compared with bortezomib-responsive patients, implying that CXCR4 mediates chemo-resistance in MM. In summary, the experimental results indicate that the CXCL12/CXCR4 axis mediates intercellular coupling, indicating that myeloma microhabitats could be used as targets for improving and developing therapeutic approaches.

In conclusion, the role of mitochondrial trafficking in BM microenvironment is crucial for the pathogenesis, progression and drug resistance of hematological tumors (Table 1). Most of the current studies on mitochondrial transport involve hematological malignancies such as AML and ALL. In fact, mitochondria can also be regarded as potential targets for treatment in other hematological diseases such as CLL. For example, a study found that changing the apoptosis-dependent character of mitochondria restored the susceptibility of CLL cells to chemotherapy [162]. In the future, mitochondrial transfer is expected to be found between different cells of more hematological benign and malignancies.

**Table 1:** Mitochondrial transfer studies in hematological malignancies

Donors	Recipients	Mechanisms of transport	Triggers	Cellular Effect	Reference
BMSCs	AML	endocytosis	Chemotherapy agents	less sensitive to chemotherapy	[131]
BMSCs	AML	TNTs	NOX2-derived ROS	Increased mitochondrial respiration and ATP production	[22]
BMSCs	AML	TNTs	OXPHOS suppression	secondary resistance to chemotherapy	[134]
BMSCs	CML	TNTs	ND	Increased survival and chemo-resistance	[143]
T-ALL	BMSCs	TNTs, ICAM-1	Chemotherapy agents	Induction of drug resistance	[146, 148]
BMSCs	ALL	TNTs	ND	Increased viability and resistance	[153]
MSCs	MM	TNTs	Cx43	increased drug resistance	[157]

ND: not defined; AML: acute myeloid leukemia; T-ALL: T cell acute lymphoblastic leukemia; ALL: acute lymphoblastic leukemia; MM: multiple myeloma; TNTs: tunneling nanotubes; BMSCs: bone marrow stromal cells; OXPHOS: oxidative phosphorylation; ROS: reactive oxygen species.

## Conclusion and perspectives

Here, we offer an overview of intercellular mitochondrial transfer, and discuss the modes and mechanisms underlying the process in diverse illnesses. Mitochondrial transfer has multiple different effects. The main reason is that mitochondria with reduced membrane potential can be released from cells of injured tissues, thus maintaining cellular homeostasis. In addition, mitochondria can also rescue metabolically damaged neighboring cells more altruistically, so as to promote the repair of cellular damage in a variety of tissues and apply to the treatment of many diseases. At the same time, cancer cells receive mitochondria from their neighbors to restore energy metabolism and increase proliferation, invasion and metastasis as well as resistance to chemotherapy. The above evidence suggests that mitochondria could be used as therapeutic targets in future therapy. This knowledge raises significant potential for modulating mitochondrial transfer to enhance normal cellular homeostasis and energy dynamics or to disturb the pathologic adaptations of tumor cells.

To date, it has been found that intercellular mitochondrial transfer plays a major role in the tumorigenesis, progression and drug resistance of hematological malignancies like AML and ALL. In other hematological malignancies, mitochondria are also targeted to enhance sensitivity to chemotherapeutic agents. All these findings could pave the way for a novel approach to the treatment of hematological malignancies in days to come. The current treatment by blocking TNTs can inhibit mitochondrial transfer and has worked well. Although the mechanism of mitochondrial transport is still not well understood, it may be related to key molecules such as CD38, motor protein KIF5B, and Cx43. These molecules could provide molecular targets for targeted therapy of hematological malignancies. At the same time, the key challenge relating to mitochondrial transfer therapy is their dual role in normal hematopoiesis and malignant diseases. As mentioned earlier, BMSCs under stress conditions provide mitochondria to HSCs. Therefore, during the targeting mitochondrial transfer therapy, it is of vital importance to consider the specific selection of recipients of mitochondrial transport between HSCs and hematopoietic malignant cells as well as the accurate regulation of the direction of transfer. It is not yet known whether the transfer of mitochondria from stromal cells to HSCs or malignant cells will have any effect during chemotherapy. Moreover, in normal hematopoiesis and malignant diseases, the differences in the intensity or types of the stress, as well as the diversities in the BM stromal microenvironment may have an effect on the bias of mitochondrial transfer. Therefore, when blocking mitochondrial transfer for hematological malignancies therapy, we should try to avoid the impact of off-target effects and make effort to develop more specific targeting molecules.

Nonetheless, additional research is required to cross knowledge gaps, eliminate clinical application challenges, and address ethical concerns related to the therapy.

## Data Availability

The datasets used during the current study are available from the corresponding author upon reasonable request.
